# Accelerating systems biology research and its real world deployment

**DOI:** 10.1038/npjsba.2015.9

**Published:** 2015-09-28

**Authors:** Hiroaki Kitano

**Affiliations:** 1 Editor-in-Chief, The Systems Biology Institute, Tokyo, Japan

Almost two decades have passed since the inception of the modern form of systems biology. Today, systems-oriented research is the mainstream approach and has been successfully applied in drug discovery, biotechnology and the clinic.

*npj Systems Biology and Applications* was created to further accelerate research in this field and its real world deployment. With a partnership between Nature Publishing Group and the Systems Biology Institute, we aim to provide a key publication platform and a major forum for discussion for those who are interested in systems-oriented research in biomedical sciences, healthcare, biotechnology and much broader area of study and their applications into business and medical practices.

It is interesting to note that there are still divergent views on systems biology due to multiplicity of approaches that range from bottom-up detailed modeling to large-scale ‘omics’ approaches, through to biomedical – top–down – approaches and engineering approaches. This divergence does not come as a complete surprise as there are a number of approaches to understanding system properties of biological systems depending on the scientific question and available analytical techniques. Nevertheless, at the core of the field lies the need to understand biological systems as ‘systems’ not just one or more of its components or just a components’ list. Our ambition may be to arrive at fundamental principles of biological systems through multiple approaches each uncovering a part of complex biological reality. Therefore, the journal embraces broad approaches that deserve publication.

Some approaches may seek to discover how systems biology can be integrated into distributed networks and devices that are ubiquitously deployed in the real world. In healthcare, an example would be a mobile phone or ambient devices that monitor and assist people’s health, disease prevention and detection. An alternative example would be a densely integrated system that spans medical records through to experimental devices that can handle data from diagnostic devices, behavioral monitoring, genome analysis, metabolic and proteomics analysis, and other basic and clinical data assets in highly integrated manner. This is systems biology embedded in the global network of devices and mobile world. Systems biology will have a critical role in making sense of data generated from such unprecedented level of connectivity.

As current resurgence of systems biology moves into its third decade, I encourage the community to tackle a new and fundamental challenge. The challenge is how to overcome our cognitive limitations for understanding and communicating reality of biological systems and data from multiplex, extremely high dimension, non-linear and dynamical systems. Experimental data and public databases are not error-free and tend to be very noisy. Our language-based communication is one of fundamental limitations. Language inevitably reduces the world to words that represent some concepts or aspects of phenomena that the observer has recognized. This cognitive process inevitably involves inaccuracy. We are also inefficient in handling large volumes of data and information that are generated everyday. Vast data and knowledge is generated in biomedical domains without being properly processed, decoded and integrated as a consistent body of knowledge. In this regards, I consider scientific discovery in biomedical science still to be at the level of cottage industry, hence the need for industrial revolution in biomedical discovery. Recent progress in Artificial Intelligence (AI) has begun to address this issue, although we are a long way off from creating an engine of scientific discovery. My own experience in AI research leads me to believe that the day will come when AI will surpass human capability in biomedical research. At least, the day will come when we will need to work with AI-based scientific discovery system for modernized scientific activities. Although this may sound like Sci-Fi to some I would like to encourage studies on such a grand challenge. Yes, it is a moonshot; however, I believe it is one of the missions of this journal to encourage bold ideas to be seriously examined.

*npj Systems Biology and Applications* encourages submission of work that not only demonstrates success and maturity of the systems biology, but also represents out-of-the-box ideas, new challenges and work that stretches the traditional ‘systems biology’ boundaries.

